# Application of Microbiological Screening Tests in Assessment of Environmental Exposure to Antibiotics: Preliminary Studies

**DOI:** 10.3390/jox14030067

**Published:** 2024-09-04

**Authors:** Daria Madej-Knysak, Ewa Adamek, Leon Kośmider, Wojciech Baran

**Affiliations:** Department of General and Analytical Chemistry, Medical University of Silesia, Jagiellońska 4, 41-200 Sosnowiec, Poland; dmadej@sum.edu.pl (D.M.-K.); eadamek@sum.edu.pl (E.A.); leon.kosmider@gmail.com (L.K.)

**Keywords:** antibiotics, bioassay, microbial toxic concentration, environmental pollution, microbial drug resistance

## Abstract

Contact of aquatic microbiocenoses with antibiotics present in the environment can cause the former to develop resistance to antimicrobial drugs. Therefore, the search for methods to detect antibiotics and drug-resistant microorganisms in the environment is important. The presented paper proposes a simple procedure to assess environmental exposure to antibiotics and the presence of non-susceptible microorganisms. Medium solutions with selected antibiotics and a microbial growth indicator were applied to test plates, and were inoculated with water samples from various ecosystems. After incubation, the susceptibility of the microorganisms to antibiotics was determined and presented in chronic microbial toxic concentration (MTC) values. It was confirmed that the presented procedure enables the assessment of the antibiotic susceptibility and adaptation potential of unselected microorganisms from different aquatic ecosystems. However, the MTC values depend on the inoculum volume, the density and seasonal activity of the microorganisms, the method of inoculum preparation, and the incubation time of the test plate. The described procedure may be practically applied as a screening test to identify the presence of drug-resistant microorganisms. Additionally, it may also be suitable as a method to assess environmental exposure to antibiotics. However, prior standardisation is required before implementing this procedure in quantitative studies.

## 1. Introduction

The widespread use of antibiotics has significantly reduced mortality caused by bacterial infections. This has also had a positive impact in terms of increasing the efficiency of livestock farms. The use of antibiotics has helped to keep livestock herds healthy and has further stimulated an increase in meat production. However, frequent antibiotic use has also resulted in microorganisms developing a defence reaction—drug resistance. According to Larson and Flach [1], the natural production of antibiotics in the environment most likely contributed to the evolution of antibiotics resistance genes (ARGs); however, this was not responsible for the rapid evolutionary expansion and spread of resistance factors across strains, species, and environments that we have observed since the introduction of antibiotics as therapeutic agents. Nowadays, the occurrence of drug resistance to all antibiotic groups used therapeutically is observed [1,2,3,4,5,6]. Cross- or multi-drug resistance also frequently occurs [6,7]. According to estimates, drug-resistant pathogens were responsible for the deaths of 1.27 million people in 2019 alone, but they may cause as many as 10 million human deaths per year by 2050 [8]. Concurrently, the emergence risk of so-called superbugs is increasing. Superbugs are resistant not only to antibiotics, but also to routinely used disinfection methods. There are new drugs in development and under clinical testing that promise no resistance generation [9,10]. On the other hand, according to Larson and Flach [1] “The most striking feature of the environmental microbiome is its immense diversity, providing numerous genes that potentially could be acquired and used by pathogens to counteract the effect of antibiotics. All approved antibiotic classes so far have been met by resistance in at least some of the pathogens they target. This suggests that external environments already harbor resistance factors for all antibiotics that will ever be developed”. Modern research indicates that a significant contribution to the development of antibiotic antimicrobial resistance derives from the use of these drugs in livestock farms. Almost 100% of tested samples of animal origin contain antibiotic resistance bacteria (ARB). Antibiotic resistance genes (ARGs) are detected in meat and livestock faeces [4,11,12,13,14]. Incidentally, livestock waste can contain antibiotics at concentrations higher than those recognised as therapeutic. The described examples of extremely high concentrations of antibiotics in livestock waste are shown in Table 1.

As a result of the release of livestock waste into the environment, the supply and diversity of ARGs is increasing rapidly [14]. Li et al. [23] reported that only 3 ARGs were identified in unfertilised soil, while 15 and 17 ARGs were detected in soil fertilised with manure from dairy cattle and poultry farming, respectively. Furthermore, antibiotics, their metabolites, and biological material with ARGs are introduced into the environment due to the inefficient treatment of wastewater or by microorganisms living in sewers [24,25,26].

Wastes containing antibiotic drugs disperse rapidly in the hydrosphere, reaching concentrations of nanograms per litre [27,28].

These concentrations are much lower than those causing toxic effects [1,29,30,31,32,33]. Therefore, the currently used environmental risk assessment systems do not indicate any environmental risk associated with the presence of low concentrations of antibiotics in aquatic environments [1,34]. On the other hand, Thiele-Bruhn and Beck [35] showed that tetracyclines and sulphonamides affect the physiological processes of soil microorganisms at concentrations several orders of magnitude lower than those considered toxic. Of particular concern is the fact that trace amounts of antibiotics were detected in almost 100% of the samples analysed. Furthermore, these drugs are found in samples from strictly protected areas in Tatra National Park in Poland [36] or virtually uninhabited areas (Antarctica) [37]. 

Many scientists point out that environmental pollution due to antibiotics increases the risk of antibiotic resistance developing [1,2,3,6,24,27,38,39]. The aquatic environment is indicated as an ideal setting for the acquisition and dissemination of antibiotic resistance. This may pose additional risks to human health [2,7] 

The mechanism of this phenomenon is not clearly explained [3,40]. A hypothesis on the influence of subinhibitory concentrations of antibiotics on microbiocenosis was described, among others, by Bernier and Surette [3]. They indicated, among others, the possibility of gene expression in even trace amounts of antibiotics. Resistance genes can spread in the environment, for instance by horizontal transfer [1,6,41]. Therefore, biocoenoses exposed to very low concentrations of antibiotics may show increased tolerance to these drugs. Isolates from stool samples form Guarani Indians, with little contact with modern pharmacotherapy, were reported to have resistance to sulphonamides and tetracyclines [42].

The presence of high amounts of antibiotic-resistant microorganisms in water samples can indicate contamination of the ecosystem by sewage, field runoff, etc. The implications related to the presence of antibiotics in an ecosystem may also manifest long after the physical drug’s removal [1,2]. Therefore, monitoring the occurrence of ARGs appears to be a much more effective method for assessing the antibiotic exposure of an ecosystem than performing direct analysis, e.g., using physicochemical methods, including HPLC-MS.

Rapid antibiotic resistance assessment kits based on PCR tests are commercially available. However, these kits are not applicable to screening tests due to their high cost and specificity.

The aim of our study was to develop a simple and inexpensive screening test to assess environmental exposure to selected antibiotics. We employ an adaptation of the microbial assay for risk assessment (MARA) methodology described by Gabrielson et al. [43] and Wadhia et al. [44,45]. This bioassay is applied to assess chronic toxicity, and uses ten bacteria of diverse taxonomies and yeast as bioindicators. The modifications to the MARA bioassay that we implemented include the following: (1) the use of environmental samples as inoculum in place of bioindicators of known taxonomy, and (2) assessment of the growth inhibition of unselected microorganisms in environmental samples in the presence of a concentration gradient of antibiotics.

The bioassay modified via this approach has been successfully applied to assess the effectiveness of advanced oxidation processes used to degrade antibiotics [46,47].

## 2. Results and Discussion

**Figure 1:**
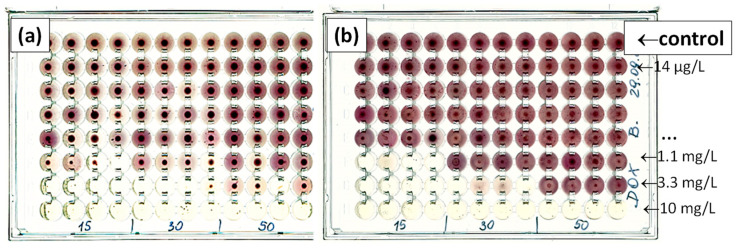
Scans of test plates containing DOX (C_max_ 10 mg/L) inoculated with different volumes of inoculum from Przemsza River (**a**) or Brynica River (**b**). Incubation duration: 24 h; temperature: 303 K.

To observe the antibiotic-induced effects on the microbiocenoses studied, cultures were prepared in 96-well U-bottom test plates with tetrazolium red as a dye and with soya peptone medium. The dye was reduced in the mitochondria of living cells to deep-red, water-insoluble formazan. The resulting product accumulated at the bottom of the test plate wells. Formazan quantity was directly proportional to the metabolic activity of the microorganisms, and corresponded to the pellet size observed on the test plate. Figure 1 shows exemplary scans of the test plates with a concentration gradient of doxycycline (DOX) and inoculated with water samples (15, 30 or 50 µL) from Przemsza River (Figure 1a) or Brynica River (Figure 1b). The medium in the lowest row of test plates contained DOX with a maximum concentration of 10 mg/L. In each subsequent higher row, antibiotic concentrations were three times lower. The control row contained the medium without antibiotics. Detailed information on test plate preparation and incubation is described in Section 3.3. The presented procedure provides a method for assessing the chronic effect of antibiotics on microorganisms in the inoculum.

The mean values of the chronic microbial toxic concentration (MTC, Section 3.4) were calculated via the analysis of the test plate scan. The software used in result analysis allowed us to determine the pellet sizes in the control and investigated rows as numerical values. On this basis, the inhibition of microorganism growth can be determined depending on the antibiotic concentration. The MTC values are calculated by comparing the area below and above the inhibition curve (Equation (1), Section 3.4). The MTC value is inversely proportional to the susceptibility of the microorganisms to antibiotics. Furthermore, this value is close to the lowest observed effect concentration (LOEC), which is the lowest concentration that enables the observation of statistically significant inhibition [43]. Therefore, the MTC value is a more reliable indicator of the effect of antibiotics on microorganisms than the acute values of L(E)C50. The result is quantitative information on the toxicity of the substance contained in the culture medium to the inoculum microorganisms. It is not dependent on the shape of the inhibition curve [43]. An important factor influencing the MTC value is the sensitivity of microorganisms to antibiotics, including the presence of strains with high natural resistance. However, determination of the MTC value using plate tests requires proper selection of the concentration range of the toxic substance. An inappropriate incubation time and too high a volume of inoculum are also unfavourable [43,48]. The intensity of the colour of samples or the presence of interfering substances (e.g., heavy-metal ions, bases, preservatives, strong reducers or oxidants, etc.) may affect the interpretation of results, potentially introducing errors. 

**Figure 2:**
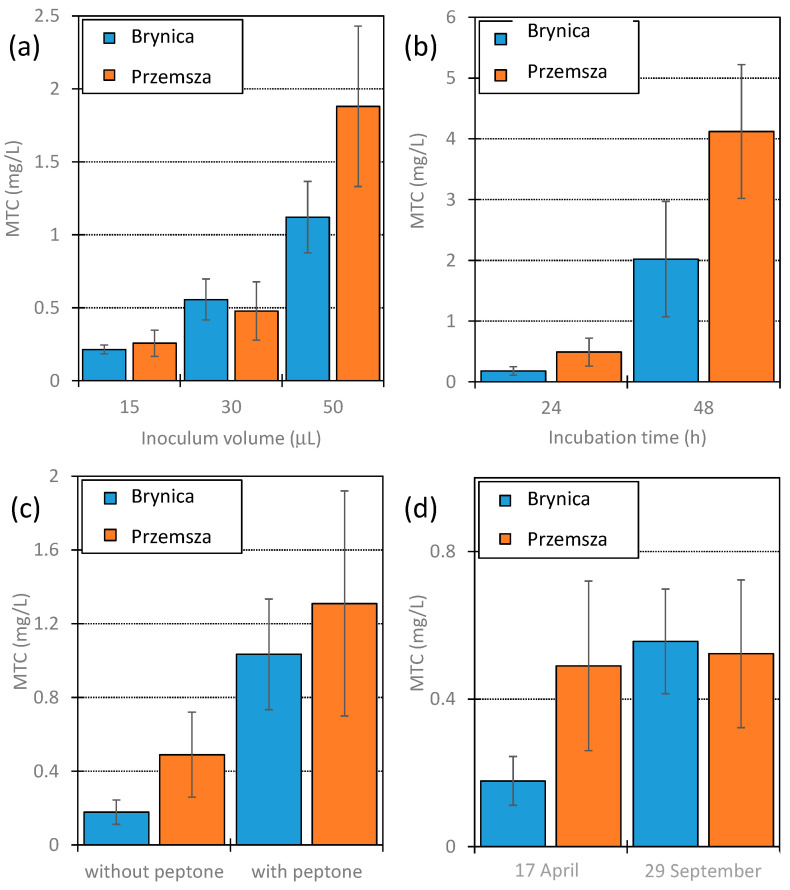
MTC values determined for DOX using inoculum from Brynica River and Przemsza River. (**a**) Effect of inoculum volume—incubation duration: 24 h; collection date: 29 September. (**b**) Effect of incubation duration—inoculum volume: 30 µL; collection date: 17 April. (**c**) Effect caused by peptone addition (50 mg/L) to inoculum and incubation for 24 h before inoculation—inoculum volume: 30 µL; incubation duration 24 h, collection date: 17 April. (**d**) Effect caused by differences in inoculum collection date—inoculum volume: 30 µL; incubation duration: 24 h.

**Figure 3:**
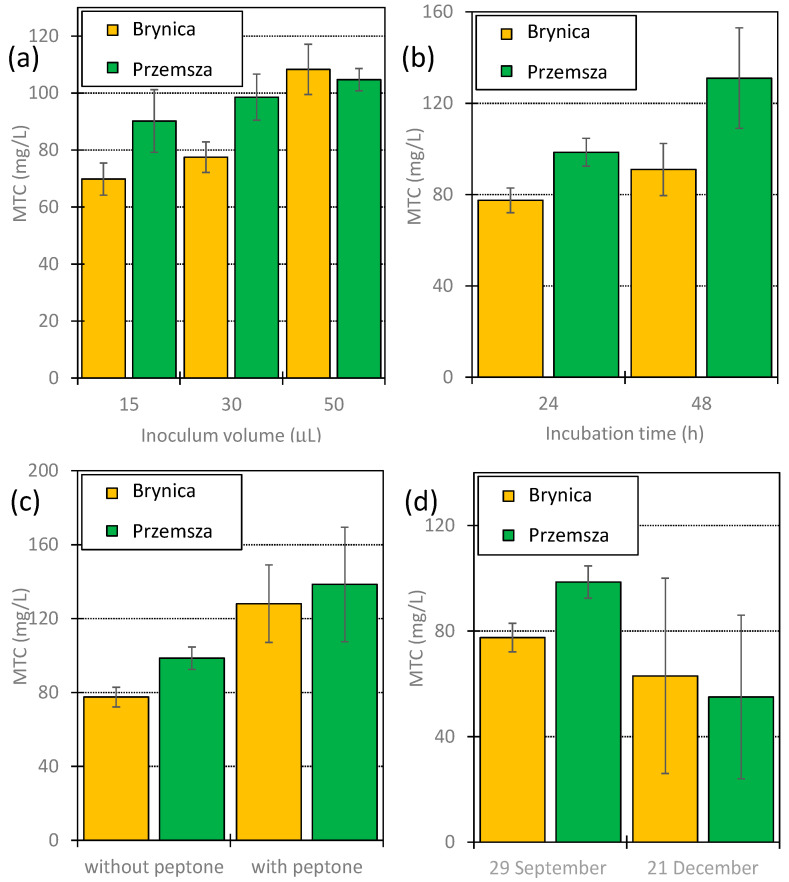
MTC values determined for TYL using inoculum from Brynica River and Przemsza River. (**a**) Effect of inoculum volume—incubation duration: 24 h; collection date: 29 September (**b**) Effect of incubation duration—inoculum volume: 30 µL; collection date: 29 September (**c**) Effect caused by peptone addition (50 mg/L) to inoculum and incubation for 24 h before inoculation—inoculum volume: 30 µL; incubation duration 24 h; collection date: 29 September (**d**) Effect caused by differences in inoculum collection date—inoculum volume: 30 µL; incubation duration: 24 h.

Figure 2 presents a comparison of the MTC values determined for DOX in the microbiocenoses of Brynica River and Przemsza River obtained at different volumes, at different sampling dates, and with different methods of preparing the inoculum, as well as for the different incubation durations. An analogous comparison for tylosin (TYL) is presented in Figure 3.

The growth inhibition of microorganisms from the studied rivers was observed via the presence of DOX and TYL at the concentrations used in the experiment. Significantly lower MTC values for DOX were observed (Figure 2). They ranged from 0.12 to 4.2 mg/L, depending on the test plate preparation conditions. These MTC values were similar to those received in the commercial MARA^®^ bioassay. The mean MTC value determined for DOX for 10 taxonomically different bacterial strains was 0.63 mg/L, while the minimum value (*D. acidovorans*) was 3.3 ± 0.1 µg/L [46,47]. On the other hand, the MTC values for TYL were significantly higher than those for DOX and ranged from 55 to 138 mg/L. The mean value for this antibiotic, as determined in the MARA^®^ bioassay, was 57 mg/L [46,47].

In all studied cases, the application of a larger inoculum volume resulted in an increase in MTC values. This relationship was markedly more progressive for DOX (Figure 2a). Increasing the inoculum volume from 15 to 50 µL yielded more than five-fold higher MTC values for this antibiotic for Brynica River and seven-fold higher MTC values for Przemsza River. The increases in MTC values for TYL for microorganisms from Brynica River and Przemsza Rivers were only 39 and 16%, respectively (Figure 3a).

A larger volume of the inoculum resulted in an increase in the abundance of microorganisms in the wells. Consequently, more formazan was formed and it was easier to read the results. However, an excessive supply of microorganisms may have meant that the observed pellet sizes were not proportional to the activity of the microorganisms. The negative influence of too large an inoculum volume on the reliability of the results was also described by Hossain [48]. In our opinion, the optimal inoculum volume on the test plate was 30 µL. Unfortunately, when an inoculum containing low amounts of microorganisms was used, the observed size of the pellets in wells in one row varied significantly.

As expected, prolonged incubation of the test plate (from 24 to 48 h) also resulted in increased MTC values. Changes in values were significantly greater for DOX (Figure 2b) than for TYL (Figure 3b), similar to the effect of the inoculum volume on the MTC values. Prolonged incubation allows inoculum microorganisms to adapt and develop less susceptible organisms. Therefore, the comparison of the results obtained after 24 and 48 h of incubation can provide valuable information regarding the adaptability of the investigated microbiocenoses. In this context, the slight increase observed in the MTC values for TYL solutions after the application of the Brynica River inoculum (Figure 3b) may suggest the low adaptation potential of the microbiocenoses of that river to TYL.

For environmental samples containing small numbers of microorganisms, e.g., in spring water, very small amounts of formazan formed may be insufficient to receive a result. In this case, sterile medium (peptone) can be added to the environmental sample. After 24 h of conditioning at room temperature, the prepared solution can be applied to the test plate. The application of the medium should lead to an increase in the abundance but not diversity of microorganisms. In practice, this effect corresponds to increasing the volume of the inoculum. It was observed that after conditioning the inoculum with peptone, the MTC values increased by several orders for DOX (Figure 2c) and to a lesser extent for TYL (Figure 3c). Most likely, the determined MTC values were overestimated in our case. Therefore, in our opinion, water samples should be analysed as soon as possible after sampling.

In temperate climates, significant seasonal changes in microbiosphere activity are observed. In general, microbial activity is the highest in summer and early autumn and decreases drastically during the winter season [49]. Therefore, we also compared the MTC values obtained for water samples taken for DOX experiments in late winter and early autumn (Figure 2d), and for TYL experiments in early autumn and early winter (Figure 3d). 

A measure of organic compound (biomass) content in water that is used in ecology is total organic carbon (TOC) [50]. This value was determined via the Lange method using commercial cuvette tests (Section 3.2). The studied samples exhibited no significant differences in total organic carbon (TOC) values. Only the inoculum collected from Przemsza River in late winter and early autumn did not show changes in activity. In all other experiments, it was confirmed that seasonally greater microbial activity (also biodiversity) resulted in higher MTC values (Figure 2d and Figure 3d). 

**Figure 4:**
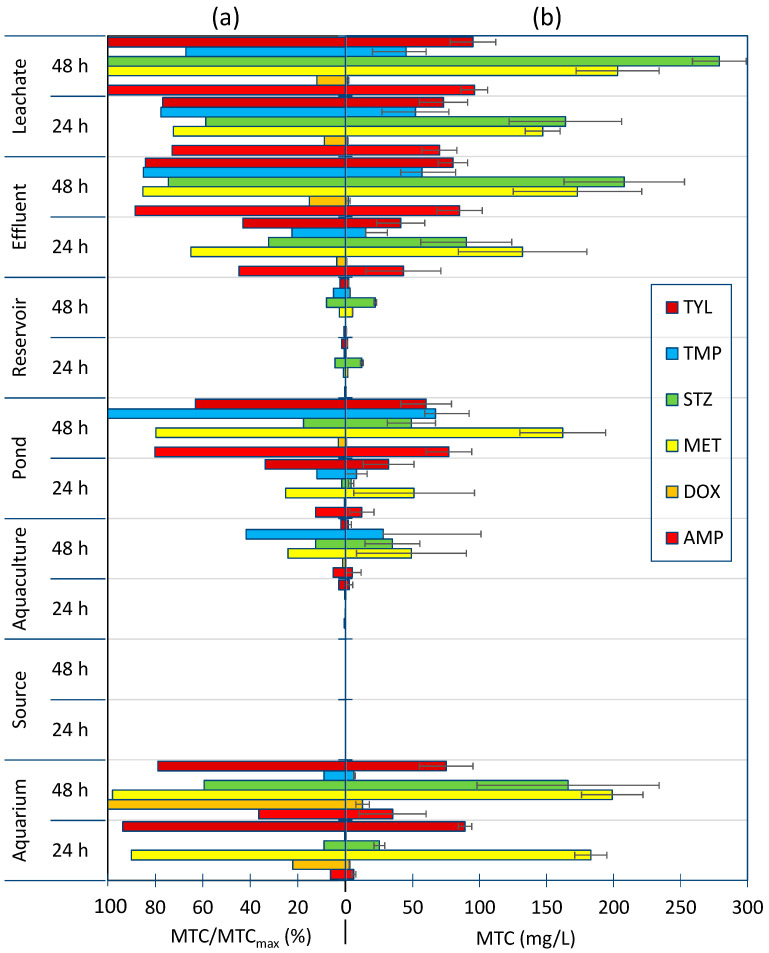
Relative MTC values (**a**) and absolute MTC values (**b**) determined after 24 and 48 h of incubation for test plates containing a gradient of antibiotic concentrations and inoculated with 30 µL of the studied environmental samples.

Ampicillin (AMP), DOX, metronidazole (MET), sulphathiazole (STZ), trimethoprim (TMP), and TYL were selected for experiments to confirm the association of MTC values with previous exposure of the microbiocenoses to antibiotics. For this purpose, seven water samples with various origins were also tested:

- Water collected from an aquarium (Figure 4, Aquarium) with a controlled culture of *Carassius auratus*;
- Water collected directly from a source (Figure 4, Source), free of anthropogenic pollution;
- Water collected from ponds with *Salmo irideus* and *Acipenser baerii* culture (Figure 4, Aquaculture) directly fed with spring water, where no antibiotics were used in the culture;
- Water from an undrained pond (Figure 4, Ponds) stocked with fish and used by anglers in a park;
- Water from an artificial reservoir (Figure 4, Reservoir) directly fed with spring water and used for recreation in the summer season;
- Effluent from the mechanical–biological wastewater treatment plant (Figure 4, effluent) located in Sosnowiec-Zagórze (Poland)supplied mainly with municipal and hospital wastewater;
- Water from a drainage ditch (Figure 4, Leachate) fed with leachate from vegetable gardens and domestic wastewater from several residential buildings.

The sampling location and water characteristics are detailed in Section 3.2. All samples were collected in early spring and used immediately (without conditioning).

The MTC values obtained are shown in Figure 4b. As the results on the microbial susceptibility to the antibiotics selected for the study are considerably different, the MTC values determined for them are also different. To support the interpretation of the results, the relative MTC values are also presented in relation to the highest value (MTC_max_) for each drug studied (Figure 4a). In each case, 30 µL of inoculum was applied to the first eleven test plate columns and then incubated at 303 K for 48 h. The twelfth column was treated as a control for medium contamination. The plates were scanned after 24 and 48 h.

To the controlled culture of *C. auratus*, MET was added once at a concentration of 20 mg/L. This drug is used in aquaristics and is slowly biodegraded in water [51]. The aquarium was equipped only with an internal sponge filter. For the 2 months preceding sampling, 20% of the aquarium water was changed every 3 days (disinfected and settled water was added). A high TOC value of 18.1 ± 0.9 mgC/L was measured, as the bred fish heavily contaminated the aquarium.

As predicted, after 24 h of incubation, a notably high MTC value was found for MET (Figure 4). On the other hand, an unexpectedly high MTC value was observed for TYL. This antibiotic is used in commercial fish farms [52]. Thus, we cannot exclude its use during fry breeding. After 48 h of incubation, MTC values increased significantly for almost all studied antibiotics (except MET and TYL). This may indicate the high capacity of the aquarium microbiocenoses for rapid adaptation.

No microbiological activity was observed in the spring water sample after 24 and 48 h. This confirms that the spring water was free of biological contamination. Very low MTC values were also recorded in the aquaculture and reservoir samples incubated for 24 h. It is assumed that these water reservoirs were not exposed to antibiotic contamination. However, for aquaculture, the tolerance of the microbiocenoses to synthetic antibiotics used in fish breeding, MET, STZ and TMP, increased after 48 h of incubation.

Microorganisms with reduced susceptibility to AMP, MET, TMP and TYL were already present in the pond water sample after 24 h of incubation. The prolonged incubation of this sample resulted in a significant increase in MTC values for the antibiotics mentioned above. This may confirm that the pond microbiocenoses were exposed to the antibiotics. The sources of antibiotics and/or ARGs may have been from regular fish stocking or even anglers’ activities.

It is undeniable that the effluent and leachate microbiocenoses were exposed to antibiotics. This is supported by the resulting data. The MTC values for the antibiotics studied (excluding DOX) incubated with effluent and leachate already achieved high values after 24 h of incubation. Moreover, for leachate incubated for 48 h in the presence of AMP, MET, STZ, and TYL, the highest MTC values were compared with those from the other experiments (Figure 4b). 

The presented results show the relationship between the determined MTC values for the inoculum microbiocenoses and the potential exposure of the ecosystem to antibiotic contamination. Therefore, the proposed procedure could be applied as a versatile and inexpensive screening test. It may also provide preliminary information on the drug resistance of microorganisms prior to the identification of ARGs. However, this conclusion requires additional verification with genetic tests. Nevertheless, the presented procedure for assessing the microbiosphere’s susceptibility to a selected antibiotic indicates the presence of microorganisms characterised by increased tolerance to that drug. Importantly, this procedure is not limited to one mechanism of antimicrobial resistance and includes the potential possibility of developing a drug resistance mechanism other than that described to date. Furthermore, prolonging the incubation time and re-scanning the plate after 48 h provides information on the potential for adaptation of the investigated microbiosphere. 

## 3. Materials and Methods

### 3.1. Antibiotics

The characteristics of the drugs used in the study are shown in Table 2.

### 3.2. Water Samples Used as Inoculum

The characteristics of the water samples used as inoculum in the study are shown in Table 3. All samples were collected directly into sterile vessels and used immediately in the experiments. For studies on the effect of medium addition to water samples, the 5 mL/L of 10% sterile peptone solution (peptone from soybean meal, enzymatic digest, Sigma Aldrich, St. Louis, MO, USA) was used, and the resulting mixture was conditioned for 24 h at room temperature.

The TOC values of the water samples in Table 3 were determined using the LCK385 cuvette test (HACH LANGE, Loveland, CO, USA). The results were read on a DR 3900 spectrophotometer (HACH LANGE, Loveland, CO, USA).

### 3.3. Procedure for Preparation of Test Plate

**Figure 5:**
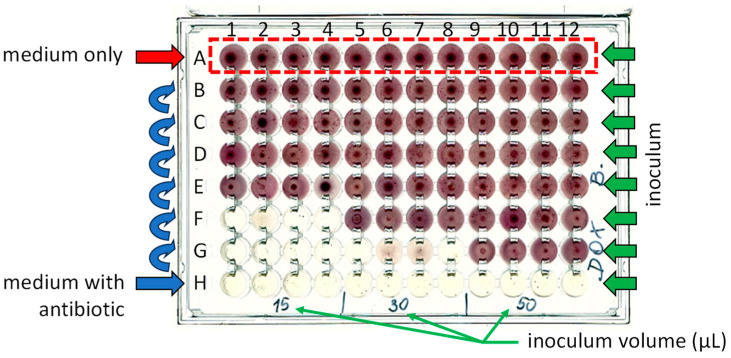
Procedure for the preparation of the test plate.

Two types of sterile, aqueous medium solutions were prepared to perform tests on the microorganisms’ susceptibility to antibiotics. The first solution (M1, medium with antibiotic, Figure 5) contained 2% soy peptone, 0.02% dye—2,3,5-triphenyltetrazolium chloride (TZR, ≥98.5%, analytical grade, POCH, Poland)—and an antibiotic of a determined concentration (Table 2). The second solution (M2, medium only) contained only peptone (2%) and dye (0.02%) without antibiotics. The prepared solutions were sterilised via filtration (sterile syringe filter CA 0.20, LLG GmbH, Germany) before application to the test plates. 

Under aseptic conditions, 150 µL of medium with antibiotic (M1) was added to each well of row H of the sterile test plates (Figure 5) using a 12-channel automatic pipette (Transferpette^®^—12 electronic, Brand GmbH, Wertheim, Germany). In the wells of rows A–G, 100 µL of antibiotic-free medium (M2) was added. Then, 50 µL of M1 was transferred from the wells in row H to the corresponding wells in row G, and the contents were mixed thoroughly. Proceeding similarly, 50 µL of the contents of the wells of row G were transferred to row F and mixed. The procedure was repeated up to row B, thus obtaining a dilution gradient of antibiotics with a step of 3. To maintain the same volume of solutions in all wells, 50 µL of solution was taken from the wells in row B and discarded. The medium without antibiotics was placed in the wells of row A. This row was a control for microbial growth. The test plate was then inoculated with water samples measuring 15 µL, 30 µL, and 50 µL. The prepared test plates were incubated for 48 h at 30 °C. After 24 and 48 h of incubation, the test plates were scanned (HP Scanjet G4050).

### 3.4. Assessment of Microbial Susceptibility

Scans of the test plates were analysed using MARA^®^ ver.1.0.0.0 software (NCIMB Ltd., Scotland, UK). The sizes of pellets in the wells of control row A (P_0_) and of pellets in the wells of rows B–H containing antibiotic medium (P_i_) were determined. Subsequently, the MTC values were calculated, and are expressed via the following formula (Equation (1)):

(1) $${\mathrm{MTC}=C}_{\min}{\times d}^{\left(\frac{P_{\mathrm{tot}}}{P_{0}}\right)-1}$$

where: C_min_ is the lowest concentration of the studied antibiotics and d is the dilution step. P_tot_ is the sum of the pellet sizes (P_i_) in rows B to H (Equation (2)):

(2) $$P_{\mathrm{tot}}=\sum_{B}^{H}P_{i}$$

Each column of the test plates was analysed separately. The plots show the mean values of the MTC along with the standard deviation. The MTC values shown in Figure 2 and Figure 3 are the mean and standard deviation of eight measurements (two plates × four columns). The MTC values shown in Figure 4 are the mean and standard deviation of 11 measurements (1 plate × 11 columns), because column 12 on the plate was used as a purity control. 

## 4. Conclusions

The procedure using 96-well test plates was confirmed to allow for the assessment of the antibiotic susceptibility of unselected microorganisms from different aquatic ecosystems. Moreover, estimation of the adaptation potential of the investigated microbiocenoses was feasible as a result of the prolonged incubation of the test plates.

The determined MTC values depended on the susceptibility of the microorganisms to antibiotics, as well as on the volume of inoculum, the density of the microorganisms (the abundance of microorganisms in the applied inoculum portion), the prior addition of medium to the inoculum, and the incubation time of the plates. Additionally, it was confirmed that the microorganisms exhibited variable susceptibility that depended on the season.

The relationship between the occurrence of ecosystem exposure to antibiotics and our procedure results on an artificial, model aquarium ecosystem and selected environmental ecosystems was also confirmed. Thus, the presented method enables the determination of the effect of antibiotics on the microbiocenoses of the selected aquatic environment even after the physical drug is no longer present. Crucially, the results obtained are not related to the type of mechanism responsible for the microorganism’s resistance. 

The procedure presented in this paper is in the preliminary research stage. To establish its reliability, much broader studies and confirmation of the results with genetic tests are necessary. The practical application of the procedure for assessing the presence of drug-resistant microorganisms in screening tests is feasible. The obtained result of the plate test may constitute a basis for performing further tests, e.g., for the presence of ARGs. However, prior standardisation of the test is required. This should include incubation times, optimal antibiotic concentration ranges, and inoculum volumes. Too small an amount of microorganisms in the inoculum will negatively affect the accuracy of the results.

## Figures and Tables

**Table 1:** Maximum concentrations of selected antibiotics in manure.

Antibiotic Group	Antibiotic	Concentration (mg/kg Dry Weight)	Matrix	Year	Reference
Imidazole	Furazolidone	~0.3	-	2015	[15]
Macrolides	Tylosin	8.1	Beef manure stockpile	2008	[16]
	Tilmicosin	18.93	Swine manure	2023	[17]
Sulphonamides	Sulphadiazine	91	Turkey manure	2007	[18]
	Sulphamethazine	167	Swine manure	2007	[18]
	Sulphamerazine	16.50	-	2017	[19]
	Sulphadimethoxine	395.73	Calf manure	2007	[20]
Tetracyclines	Oxytetracycline	354	Swine manure	2014	[21]
	Tetracycline	136.00	Swine manure	2011	[22]
	Chlortetracycline	764.4	Swine manure	2011	[22]
	Doxycycline	99.198	Poultry manure	2023	[17]
Trimethoprim	Trimethoprim	17.0	Turkey manure	2007	[18]

**Table 2:** Characteristics of the investigated antibiotics.

Antibiotic Classification	Name	Abbreviation	Molecular Weight (g/mol)	Manufacturer/Supplier	Purity	Maximum Concentration Used in Experiments ^1^ (mg/L)
Penicillins	Ampicillin sodium salt	AMP	371.4	Sigma-Aldrich	91.0–100.5%	100
Tetracyclines	Doxycycline hyclate	DOX	512.9	Sigma-Aldrich	≥98%	10 or 50
Imidazole derivatives	Metronidazole	MET	171.2	Fluka	≥98%	200
Sulphonamides	Sulfathiazole sodium salt	STZ	277.3	Sigma-Aldrich	>99%	300
Trimethoprim	Trimethoprim	TMP	290.3	Sigma-Aldrich	≥98%	100
Macrolides	Tylosin tartrate	TYL	1066.2	Sigma-Aldrich	potency: ≥800 units/mg	200

^1^ These concentrations were determined in preliminary studies via the susceptibility assessment of microorganisms from Przemsza River.

**Table 3:** Characteristics of the investigated water samples.

Abbreviated Name	Brief Description	Geographical Coordinates of the Sampling Location in Decimal Format	Sampling Month	TOC (mgC/L)
Przemsza	The moderately polluted Przemsza River; the catchment area includes agricultural and industrial areas	50.25903590, 19.13729930	March	10.4 ± 0.5
			September	13.5 ± 0.3
			December	13.0 ± 1.0
Brynica	The heavily polluted Brynica River; the catchment area includes an industrial area	50.25894507, 19.13701568	March	18.0 ± 0.4
			September	20.3 ± 0.3
			December	17.7 ± 0.3
Aquarium	Water from *C. auratus* culture in a 50 L aquarium, without plants, without gravel substrate, with a sponge filter, and fed with bacteria-free water	-	April	18.2 ± 0.5
Source	Water from the source of Sztoła River, free from anthropogenic pollution; metronidazole was applied in culture	50.23925120, 19.50705237	April	<3.0
Aquaculture	Water from a commercial freshwater fish farm, including *S. irideus* and *A. baerii*; ponds were fed directly with spring water, and no antibiotics were used in the culture	50.43250454, 19.18074069	April	4.2 ± 0.3
Pond	Water from a small undrained pond in the park, stocked with fish and used for recreation by anglers	50.34197738, 19.18572344	April	4.1 ± 0.2
Reservoir	Water from artificial reservoir Pogoria 3; the reservoir is fed directly with spring water and is used for recreation in the summer season;	50.35563921, 19.21286766	April	7.5 ± 0.5
Effluent	Treated wastewater from the mechanical–biological wastewater treatment plant with a sludge bioreactor; the wastewater treatment plant is mainly supplied with municipal and hospital wastewater from the Sosnowiec-Zagórze housing estate (approximately 50,000 residents)	50.30227793, 19.20080535	April	24.1 ± 0.7
Leachate	Water from a drainage ditch fed with leachate from vegetable gardens and domestic wastewater from several residential buildings	50.30592077, 19.20361633	April	>30

## Data Availability

The data presented in this study are available on request from the corresponding author. The data are not publicity available due to the very large sizes of the chromatographic files.
